# Comparison of video laryngoscope, video stylet, and flexible videoscope for transoral endotracheal intubation in patients with difficult airways: a randomized, parallel-group study

**DOI:** 10.1186/s13063-023-07641-1

**Published:** 2023-09-21

**Authors:** Tao Zhang, Kai-Yuan Zhao, Ping Zhang, Ren-Hu Li

**Affiliations:** Department of Anesthesiology, Lu’an Hospital of Anhui Medical University, Lu’an, Anhui China

**Keywords:** Intubation devices, Airway management, Intubation, Difficult airway

## Abstract

**Background:**

The 2022 ASA guidelines recommend the video laryngoscope, video stylet, and flexible videoscope as airway management tools. This study aims to compare the efficacy of three airway devices in intubating patients with difficult airways.

**Methods:**

A total of 177 patients were selected and randomized into the following three groups: the video laryngoscope group (Group VL, *n* = 59), video stylet group (Group VS, *n* = 59), and flexible videoscope group (Group FV, *n* = 59). The success rate of the first-pass intubation, time of tracheal intubation, level of glottic exposure, and occurrence of intubation-related adverse events were recorded and analyzed.

**Results:**

All patients were successfully intubated with three devices. The first-pass intubation success rate was significantly higher in Groups VS and FV than in Group VL (96.61% vs. 93.22% vs. 83.05%, *P* < 0.01), but it was similar in the first-pass intubation success rate between Groups VS and FV(*P* > 0.05). The number of patients categorized as Wilson-Cormack-Lehane grade I-II was fewer in Group VL than in Groups VS and FV (77.97% vs. 98.30% vs. 100%, *P* = 0.0281). The time to tracheal intubation was significantly longer in Group FV(95.20 ± 4.01) than in Groups VL(44.56 ± 4.42) and VS(26.88 ± 4.51) (*P* < 0.01). No significant differences were found among the three groups in terms of adverse intubation reactions (*P* > 0.05).

**Conclusions:**

In patients with difficult airways requiring intubation, use of the video stylet has the advantage of a relatively shorter intubation time, and the flexible videoscope and video stylet yield a higher first-pass intubation success rate and clearer glottic exposure than the use of the video laryngoscope.

**Trial registration:**

Chinese Clinical Trial Registry. No: ChiCTR2200061560, June 29, 2022.

**Supplementary Information:**

The online version contains supplementary material available at 10.1186/s13063-023-07641-1.

## Background

Currently, visualization techniques are becoming increasingly popular for its reduction in reducing the risk of severe intubation reactions and failed intubations. Nevertheless, both anticipated and unanticipated difficult airways still result in failed intubation, which not only puts the anesthesiologist in an embarrassing dilemma, but also threatens the life and safety of the patients, we cannot use only one device to deal with all difficult airways encountered.

The video laryngoscope, a conventional glottic visualization device, has been considered the gold standard device for transoral tracheal intubation by anesthesiologists worldwide, and its availability has led to unprecedented improvements in the field of view during intubation, success rates of tracheal intubation, shortened intubation times, and even reduced intubation-related airway injuries compared with conventional direct laryngoscopy [1–3]. However, video laryngoscopy has certain limitations in clinical practice. For example, it may not be suitable for patients with spinal cord injuries who are unable to tilt their head back or for obese patients who may have difficulty in exposing the glottis. Previous study showed a correlation between different blade angulation and difficulty in delivering the tube to the glottic opening [4]. This has led to the development of many new intubation devices based on traditional visualization devices to assist anesthesiologists in both routine and difficult airway management. Flexible videoscope and video stylet are two of these devices that have given anesthesiologists a more powerful weapon in managing difficult airways and have been defined by the 2022 ASA Difficult Airway Guidelines as an advanced airway tool for managing difficult airways; however, the guidelines mention that there is a lack of literature assessing the most effective order of equipment to use when attempting intubation of an anticipated difficult airway, as well as literature assessing which equipment is the most effective for managing a difficult airway when encountered [5]. Therefore, this study focuses on patients with difficult airways and compares the effectiveness of a video laryngoscope, video stylet and flexible videoscope in transoral tracheal intubation for general anesthesia in patients with difficult airways of EI-Ganzouri risk index (EGRI) greater than or equal to 4 and provides recommendations for the preferred device for difficult airway management.

## Method

### Patients and study design

The present study was approved by the Ethics Committee of the Lu'an Hospital of Anhui Medical University (NO.2021LL005) and written informed consent forms were signed by all the participants or their relatives before enrollment. We recruited patients from the current hospital between July and September 2022. The trial was prospectively registered at the China Clinical Trial Registration Center (www.chictr.org.cn, ChiCTR2200061560; Principal investigator: Ren-Hu Li; Date of registration; 29 June 2022).

The inclusion criteria were as follows: (1) need for transoral tracheal intubation for general anesthesia during elective surgery in our hospital; (2) ASA class I and II; (3) 18–64 years of age; and (4) EI-Ganzouri risk index (EGRI) (weight, mouth opening, thyromental distance, possibility of mandibular subluxation, Mallampati class, head and neck mobility, history of difficult intubation) greater than or equal to 4 [6]. Patients with any of the following conditions were excluded: (1) difficulty receiving mask ventilation; (2) anatomical abnormalities of the upper airway (trauma, tumor, and deformity); (3) cervical spine instability; (4) severe trauma during intubation; (5) inability to railroad; (6) inability to properly understand and cooperate with the experiment; (7) and refusal to participate in this study.

Pre-anesthesia visits were performed by anesthesiologists not involved in this study, and age, sex, ASA classification, and body mass index were assessed and recorded in all cases. Most importantly, assessment of difficult airways using the EGRI was performed, including weight, head and neck mobility, mouth opening, possibility of mandibular subluxation, thyromental distance, Mallampati classification, and history of difficult intubation.

### Anesthesia protocol

#### Anesthesia preparation

All patients fasted from food and water before surgery. In all patients, dexmedetomidine was given preoperatively intravenously over 10 min before the induction of anesthesia. The patient’s body temperature, heart rate, pulse, oxygen saturation, blood pressure, electrocardiogram, and end-tidal carbon dioxide (PETCO_2_) were recorded. A bispectral index (BIS) monitor was used to measure the depth of anesthesia. For females, a reinforced endotracheal tube with an inner diameter of 7.0 mm was used, while, for males, a 7.5-mm reinforced tube was needed.

#### Induction of anesthesia

Anesthesia was inducted by sequential injections of 0.05 mg/kg midazolam, 0.5 μg/kg sufentanil, 0.3 mg/kg etomidate, and 0.8 mg/kg rocuronium in all three groups. We monitored neuromuscular function using a train of four (TOF) stimulation pattern. Intubation took place when a fully relaxed status was reached (TOF 0/4). All intubations were performed by two anesthesiologists, both of whom were attending physicians with more than 5 years but less than 10 years of clinical experience, each intubation device was used more than 200 times after training more than 100 times on manikins.

#### Group VL

**Fig. 1:**
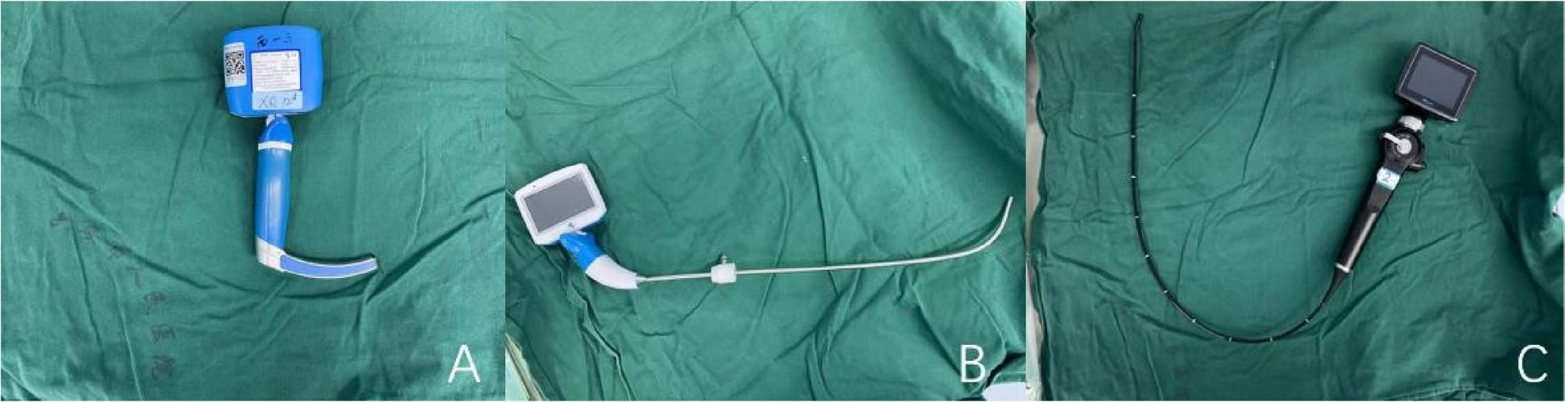
Comparison of three devices used for transoral endotracheal intubation. **A** Video laryngoscope. **B** Video stylet. **C** Flexible videoscope

The video laryngoscope (TDC- K3, UE Medical Equipment Co. Ltd., Zhejiang, China, Fig. 1A) was fitted with a disposable transparent lens jacket, and the front end of the tracheal tube was shaped to approximately 60° with the same curvature as the anterior part of the visual laryngoscope by applying a metal tube core. The lingual-palatal and palatopharyngeal arches were passed to reach the pharyngeal cavity, and when the glottic opening was exposed in the observation display, the tracheal tube was gently pushed into the trachea with the right hand, and the core was removed at the same time.

#### Group VS

The video stylet (TRS K2, UE Medical Equipment Co., Ltd., Zhejiang, China, Fig. 1B) was inserted into the tracheal tube, and the tracheal tube was fixed at the upper end to prevent the lens from sticking out of the tracheal tube and avoid contamination of the lens with secretions. After 11–12 cm (at the level of the upper larynx), the lens was aimed to the left and middle of the neck while the screen was observed to locate the glottic opening. The tracheal tube was fed into the trachea, the core was pulled out, the video stylet was withdrawn, and the capsule was inflated.

#### Group FV

The flexible videoscope (TIC, UE Medical Equipment Co., Ltd., Zhejiang, China, Fig. 1C) was inserted into the tracheal tube, the tracheal tube was fixed at the upper end of the light-guiding hose, and the patient’s jaw was lifted. Then, the light-guiding hose was placed into the patient’s mouth, and after reaching the pharynx near the glottic opening and slightly withdrawing the hose to expose the epiglottis, the front end of the device was placed in the epiglottis vallecula or below the epiglottis. The epiglottis was exposed by lifting the device up off the glottic opening. The light-guiding tube is inserted through the glottis and advanced approximately 10 to 15 cm so that it reaches the tracheal prominence. Finally, the tracheal tube was gently pushed into the trachea to 3 cm from the bulge to complete the tracheal intubation.

After successful tracheal intubation, the patient was connected to the anesthesia machine for mechanical ventilation, the tidal volume was adjusted to 10 ml/kg, and the respiratory rate 12 breaths/min to maintain PETCO_2_ at 35–45 mmHg. Anesthesia was maintained with propofol 4–6 mg/kg/h and remifentanil 0.1–0.2 μg/kg/min in each group and discontinued at the time of skin closure. This dosage was adjusted according to the measure of anesthetic depth using BIS monitoring at a target zone of 40–60. Rocuronium was used intraoperatively according to the TOF value of the muscle relaxation monitor. Sufentanil was used based on a combination of the drug’s duration of action (potency), the depth of anesthesia (BIS), changes in the patient’s blood pressure and heart rate, and the intensity of the surgical stimulation. After surgery, patients were sent to the post-anesthesia care unit (PACU).

### Patient evaluation

The success rate of the first intubation was recorded as the primary observation. The level of glottic exposure (Wilson-Cormack-Lehane grading, W–C-L) [7] and intubation time (time between the end of mask ventilation and confirmation of the waveform by end-tidal carbon dioxide monitoring) were used as secondary observations. Based on the consensus in the reviewed literature, the most appropriate method for the confirmation of successful intubation is observation of the waveform on the end-tidal carbon dioxide monitoring instrument [8, 9]. Intubation was attempted a maximum of three times. If all three intubations failed, the tube was removed, and the oxygen supply was maintained by supraglottic ventilation. The intubation device was withdrawn from patients who experienced a failed first intubation and patients were reoxygenated for 3 min before a second intubation was performed, then, the time of intubation was recorded again. Failed intubation was defined as an intubation time longer than 3 min or patient oxygen saturation reduced to less than 90%. After the intubation attempt has been abandoned, the bag-mask ventilation would be reinitiated. An additional dose of propofol (0.5–1 mg/kg) was administered, and a laryngeal mask was inserted to assist breathing. Patients were followed up in the PACU after the procedure and asked whether they had a sore throat, hoarseness, or other discomfort to record the occurrence of adverse effects associated with intubation.

### Statistical analyses

To calculate the sample size, we performed a preliminary experiment with 20 patients in each group (60 patients in total), and the first-pass intubation success rate in each group was 75% in Group VL, 95% in Group VS, and 90% in Group FV. With *α* = 0.05 and test efficacy 1 − *β* = 0.8, a minimum sample size of 155 could be calculated according to PASS15.0.5 software. To account for possible dropouts, the number of patients was increased to 177. Prior to the start of the study, all patient names were randomly placed in opaque envelopes using computer-generated random numbers according to the equipment used for intubation, by someone other than the investigator, and the envelopes containing the random numbers were divided into 3 groups. After completion of the procedure, case questionnaires containing only the random numbers were submitted by the researchers, but the followers who conducted the follow-up survey were not aware of the random groupings.

The data were analyzed using SPSS 26.0 statistical software. Continuous variables, such as the basic characteristics of patients, are expressed as the mean ± standard deviation. Comparisons of measurement data were performed using the *F* test if the conditions of normality and chi-squared were met; otherwise, the rank sum test was used. Comparisons of count data were performed using the chi-square test. All significance tests were two-sided tests with a test level of *α* = 0.05, and *P* < 0.05 indicated that the differences were statistically significant.

## Results

**Fig. 2:**
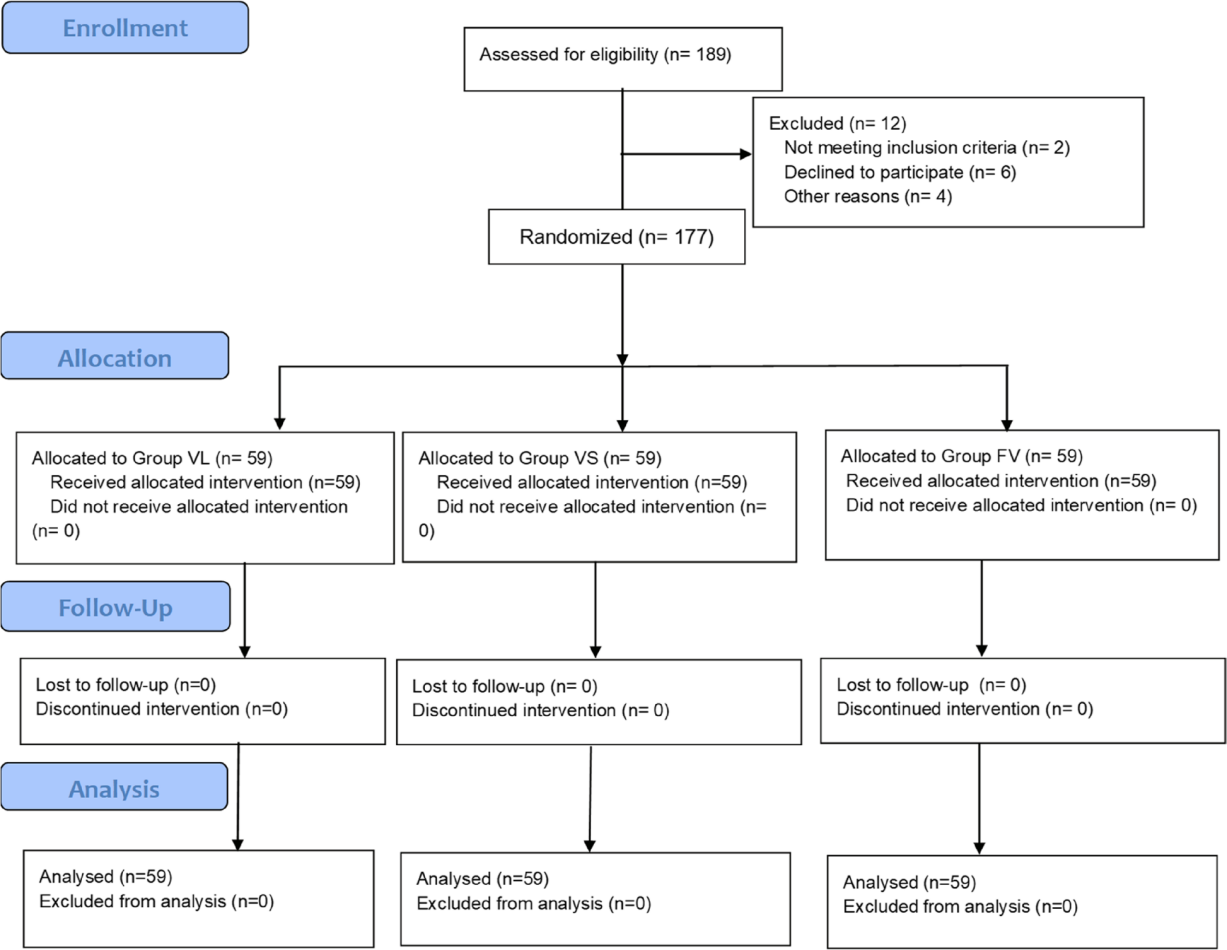
CONSORT flow chart for patient recruitment and randomization

A total of 189 patients requiring general anesthesia for elective surgery were enrolled for the present study. The CONSORT flow chart of the included patients is shown in Fig. 2. Two patients did not meet the inclusion criteria due to extremely limited mouth openings (< 2 cm), 6 patients refused to participate in the study, and 4 others were excluded due to surgical cancellation. A total of 177 patients were included, 59 in Group VL, 59 in Group VS, and 59 in Group FV. There were no statistically significant differences among the three groups in terms of sex, age, BMI, ASA classification, or El-Ganzouri score (all *P* > 0.05) (Table 1).

**Table 1:** Comparison of patient characteristics

Patient information	VL (*n* = 59)	VS (*n* = 59)	FV (*n* = 59)	*P*-value
Sex (M/F, *n*)				0.831
Male	41	39	42	
Female	18	20	17	
Age (years)	49.66 ± 5.37	50.85 ± 6.06	49.51 ± 5.42	0.369
BMI (kg/m^2^)	27.62 ± 2.24	28.04 ± 2.15	28.16 ± 2.19	0.978
ASA Classification				0.618
I (*n*)	49	51	47	
II (*n*)	10	8	12	
EI-Ganzouri risk index	6.27 ± 1.46	6.54 ± 1.33	6.32 ± 1.25	0.673

Data indicate the mean ± SD or *n*; *ASA* American Society of Anesthesiologists, *BMI* body mass index. Statistical analysis indicated no significant differences in the parameters among groups (*P* > 0.05)

**Fig. 3:**
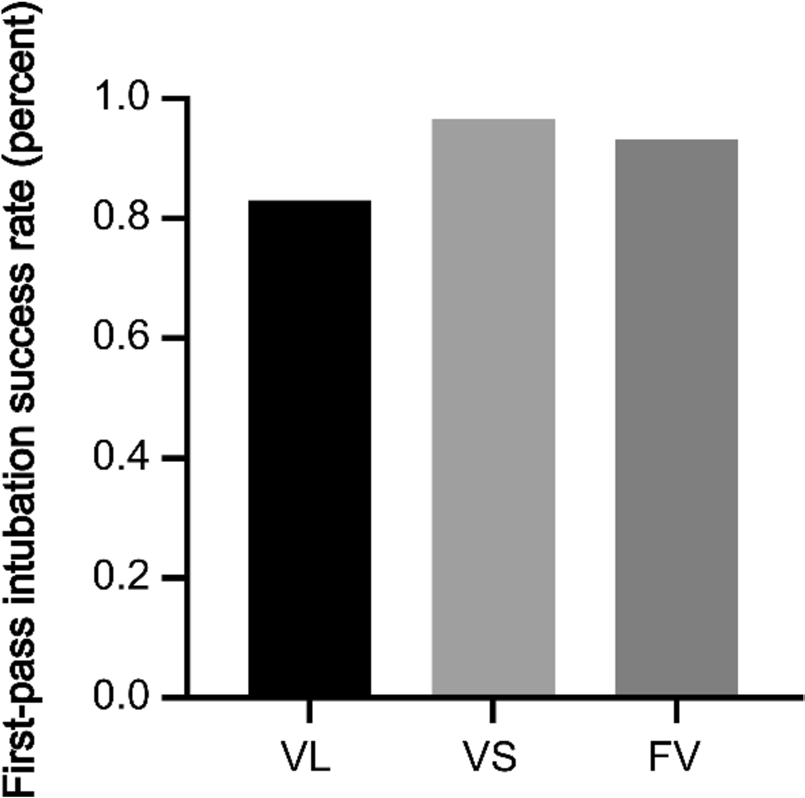
Comparison of first-pass intubation success rate

**Fig. 4:**
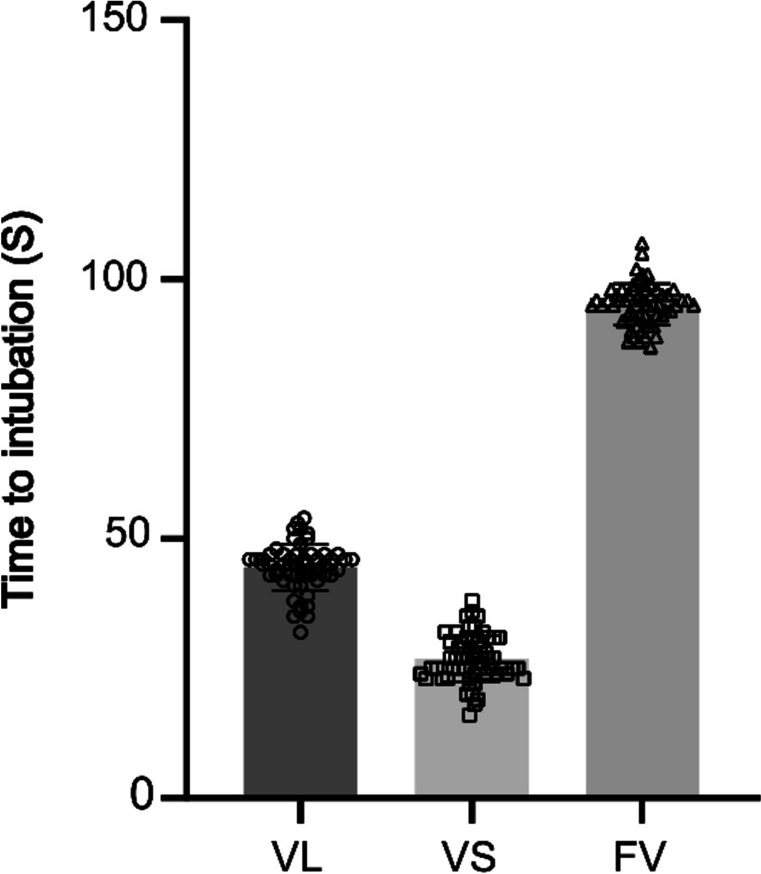
Comparison of time to intubation

The first-pass intubation success rate was significantly higher in Groups VS and FV than in Group VL (96.61% vs. 93.22% vs. 83.05%, *P* < 0.01), and the second intubation was successful in all three groups for patients whose first intubation was unsuccessful (Fig. 3). The number of patients whose glottic exposure grade I-II in Group FV and Group VS was larger than that in Group VL (*P* < 0.01), but there was not significantly difference between Group VS and Group FV. The average duration for intubation was 44.56 ± 4.42 s in Group VL, which is shorter than the duration of 95.20 ± 4.01 s observed in Group FV. Group VS exhibited the shortest average intubation time of 26.88 ± 4.51 s, indicating a notable advantage over other groups in terms of intubation time (*P* < 0.01) (Table 2, Fig. 4). There were no significant differences in the occurrence of intubation-related adverse reactions among the groups (*P* > 0.05).

**Table 2:** Comparison of acoustic portal exposure grading and intubation in each group

	VL (*n* = 59)	VS (*n* = 59)	FV (*n* = 59)	*P*-value
W–C-L classification				0.01
1, 2(*n*)	46	58	59	
3, 4(*n*)	13	1	0	
First-pass intubation success rate (*n*, percent)	49 (83.05%)	57 (96.61%)	55 (93.22%)	0.0281
Total intubation success rate (*n*, percent)	59(100%)	59(100%)	59(100%)	−
Time to intubation(s)	44.56 ± 4.42	26.88 ± 4.51	95.20 ± 4.01	0.01
Intubation-related adverse events (*n*)	11	6	4	0.1216

Data indicate the mean ± SD or *n*(percent)

*W–C-L classification* Wilson-Cormack-Lehane classification

## Discussion

In this randomized parallel-group study, we showed that all three devices were ultimately effective for intubation, higher first-pass intubation success rates were obtained with the video stylet and the flexible videoscope. Endotracheal intubation with the video stylet resulted in shorter intubation time when compared with the video laryngoscope and flexible videoscope. Glottic exposure in patients with difficult airways is a major challenge when managing the airway, endotracheal intubation with flexible videoscope and video stylet having much better glottic exposure capabilities than video laryngoscope. Clearly, for the management of patients with difficult airways, video stylet and flexible videoscope perform much better.

The increasing number of airway management devices that are being developed and used has changed the definition of a difficult airway [10, 11]. Jaime B. Hyman’s team has shown that the success rate of intubation with a conventional video laryngoscope is not ideal for patients with head and neck injuries. Most likely because the laryngeal tube is fixed to the scope, the patient’s head must be tilted back and the atlantoaxial joint must be lengthened when the scope is placed in the patient’s mouth, and these maneuvers may harm patients who need cervical bracing [12]. We found that the first-pass success rate of intubation in 177 patients with difficult airway was much lower in Group VL than in Groups VS and FV. Ten patients in Group VL failed first intubation and had the laryngeal lens withdrawn, then were reoxygenated for 3 min, had their heads tilted back more, and their mouths opened by an assistant with both hands. Thus, the intubation restriction in Group VL not only included patients with poor head and neck mobility but also included patients with difficult airways such as difficult vocal openings, which increased the intubation time and undoubtedly increased the risk of injury to the patient.

The video stylet does not require a large degree of mouth opening and head tilt and does not require the epiglottis to be lifted, which may effectively reduce the degree and force of instruments contacted with the oropharynx. Therefore, the damage to the oropharyngeal mucosa caused by intubation is reduced, which is an obvious advantage for intubation in patients with cervical bracing [13]. The study conducted by Alvis et al. indicated that the use of the video stylet in difficult airway patients who do not require awake intubation can replace a use of flexible videoscope and that time to intubation would be shorter, which is consistent with what we have observed [14]. The literature indicates that a curved video stylet will facilitate tracheal catheter delivery to the vocal hilar opening [15]. Among the 59 patients intubated with the video stylet, the total intubation success rate was 100%, but 2 patients required a second intubation because the video stylet lens was contaminated with many oropharyngeal secretions after entering the patient’s mouth, resulting in blurred visualization. Thus, the anesthesiologist did not have a clear view of the glottis, forcing him to withdraw the stylet and reintubate the patient after aspiration and reoxygenation. We aimed to overcome this limitation and found that we could obtain a very clear view by placing the lens at Murphy’s orifice of the tracheal tube without moving the lens over the tip during the present study. Even if the patient had secretions in the mouth, the video stylus was operated very smoothly to find the glottis. Although Yoon [16] concluded that superior performance could be achieved with the use of a visual laryngoscope, our experience indicated that the video stylet was easier to control and allowed confirmation of the voice portal view directly from the tip of the device without additional manipulation.

The flexible videoscope has the advantage of not limiting the diameter of the tracheal tube and, due to the softness of its light-guiding hose, is also better than a regular video laryngoscope in terms of reducing damage to the patient’s mouth and airway during intubation. However, we found that its intubation time was longer than that of the video laryngoscope and video stylet, probably because the flexible videoscope light-guiding hose was difficult to fix during the operation and it was more difficult to operate. Additionally, because muscarinic drugs and gravity reduce tension in the epiglottis and tongue, an assistant sometimes was needed to tilt the head back and elevate the jaw to open the epiglottis and obtain a view of the glottis. Tracheal intubation using a fiberscope takes longer than that with an endoscope [17]. At the same time, the use of the flexible videoscope for transoral intubation is a challenging skill for anesthesiologists to master and requires repeated practice [18]. Flexible videoscope-guided tracheal intubation has been shown to produce more dramatic hemodynamic changes [19, 20]. However, in our study, we made use of transoral tracheal intubation, whereas most previous studies have utilized transnasal intubation. The light-guiding hose has the advantage of being more flexible and smaller in caliber, and it performs better in terms of glottic exposure than the video laryngoscope. All 59 patients in Group FV in our study were classified as W–C-L grade I-II, and 4 of them were withdrawn the catheter hose after 3 attempts to avoid damage to the vocal hilum due to the resistance encountered when pushing the tracheal tube into the airway. The patient’s neck was fully tilted back, and the lower jaw was lifted; after that, the tracheal tube was reintubated and successfully delivered into the trachea. The remaining 55 patients were successfully intubated in a single attempt, and the study by Law et al. also showed that physicians skilled in the use of a flexible videoscope have a high intubation success rate in managing difficult airways [21].

This study has two limitations, as described below. Firstly, the criteria for recruiting subjects may not include all patients who meet the definition of a difficult airway. For example, in recent studies, supraglottic and subglottic ultrasound measurements or upper lip biting tests have been performed to predict a difficult airway [22, 23]. In a follow-up study, we can conduct a large-scale multicenter clinical study to confirm our conclusions. Secondly, flexible videoscope requires one hand to hold the mirror and one hand to operate the guiding hose, so an assistant is needed for opening the patient’s mouth and placing the mouth pad, which may lead to systematic errors in the three groups.

## Conclusion

When managing the airway of patients with difficult airways, all three devices can be used to achieve high intubation success rates, but the video stylet and flexible videoscope provide a better view of the vocal cords and are superior to the video laryngoscope in terms of the first-pass intubation success rate. The use of a video stylet has the advantage of a relatively shorter intubation time, allowing successful intubation in a shorter time and reducing the patient’s hypoxia time. Therefore, when anesthesiologists faced with a difficult airway, a video stylet should be the first choice, and if the airway is still not under control, a flexible videoscope should be used.

### Supplementary Information

**Additional file 1.**

## Data Availability

The datasets used and/or analyzed during the current study were available from the corresponding author on reasonable request.

## Abbreviations

VL: Video laryngoscope
VS: Video stylet
FV: Flexible videoscope
BIS: Bispectral Index Monitoring
W–C-L: Wilson-Cormack-Lehane
ASA: American Society of Anesthesiologists
PACU: Post-anesthesia care unit
PEtCO2: End-tidal carbon dioxide
